# Profile of health care workers in a context of instability: a cross-sectional study of four rural health zones in eastern DR Congo (lessons learned)

**DOI:** 10.1186/s12960-023-00816-6

**Published:** 2023-04-20

**Authors:** Charles Ruhangaza Mushagalusa, Daniel Garhalangwanamuntu Mayeri, Bertin Kasongo, Aimé Cikomola, Sammuel Lwamushi Makali, Amani Ngaboyeka, Lili Chishagala, Albert Mwembo, Abdon Mukalay, Ghislain Balaluka Bisimwa

**Affiliations:** 1 Ecole Régionale de Santé Publique, Université Catholique de Bukavu, Bukavu, Democratic Republic of Congo; 2 Hôpital Provincial Général de Référence de Bukavu, Université Catholique de Bukavu, Bukavu, Democratic Republic of Congo; 3 Centre de Recherches en Sciences Naturelles, Lwiro, Kinshasa, Democratic Republic of Congo; 4 Ecole de Santé Publique, Université de Lubumbashi, Lubumbashi, Democratic Republic of Congo

**Keywords:** Congo, Unstable health district, Healthcare center, Income, Socio-demographic profile, Health care worker

## Abstract

**Background:**

The crisis in human resources for health is observed worldwide, particularly in sub-Saharan Africa. Many studies have demonstrated the importance of human resources for health as a major pillar for the proper functioning of the health system, especially in fragile and conflict-affected contexts such as DR Congo. However, the aspects relating to human resources profile in relation to the level of performance of the health districts in a particular context of conflicts and multiform crises have not yet been described.

**Objective:**

This study aims to describe the profile of staff working in rural health districts in a context of crisis and conflicts.

**Methods:**

A cross-sectional study was carried out from May 15, 2017 to May 30, 2019 on 1090 health care workers (HCW) exhaustively chosen from four health districts in Eastern Democratic Republic of Congo (Idjwi, Katana, Mulungu and Walungu). Data were collected using a survey questionnaire. The Chi^2^ test was used for comparison of proportions and the Kruskal–Wallis test for medians. As measures of association, we calculated the odds ratios (OR) along with their 95% confidence interval. The α-error cut-off was set at 5%.

**Results:**

In all the health districts the number of medical doctors was very insufficient with an average of 0.35 medical doctors per 10,000 inhabitants. However, the number of nurses was sufficient, with an average of 3 nurses per 5000 inhabitants; the nursing / medical staff (47%) were less represented than the administrative staff (53%). The median (Min–Max) age of all HCW was 46 (20–84) years and 32% of them were female. This was the same for the registration of staff in the civil service (obtaining a registration number). The mechanism of remuneration and payment of benefits, although a national responsibility, also suffered more in unstable districts. Twenty-one percent of the HCW had a monthly income of 151USD and above in the stable district; 9.2% in the intermediate and 0.9% in the unstable districts. Ninety-six percent of HCW do not receive Government’ salary and 64% of them do not receive the Government bonus.

**Conclusion:**

The context of instability compromises the performance of the health system by depriving it of competent personnel. This is the consequence of the weakening of the mechanisms for implementing the practices and policies related to its management. DR Congo authorities should develop incentive mechanisms to motivate young and trained HCW to work in unstable and intermediate health districts by improving their living and working conditions.

## Introduction

### State of the world’s human resources for health

The crisis in human resources for health constitutes a worldwide concern. In its report on the World Health in 2006, the World Health Organization’s (WHO) has mentioned that crisis in Human Resources for Health (HRH) is observed worldwide and particularly in sub-Saharan Africa [1]. This crisis is mainly characterized by staffing problem, training profile, supervision and motivation and non-standard working conditions as well [1–4]. Developed countries also variously face problems of health workforce supply. In France, the number of health professionals in training is regulated by the health system. However, the existence of a plethora or shortage is rather linked to an unequal geographical distribution between urban and rural areas and a poor distribution between primary and secondary care specialties [5].

In most countries, the training of health professionals benefits from fairly rigorous regulation involving both the academic organizations and the government (Ministry of Health and Ministry of Higher Education), so as to ensure monitoring and control during their work. This makes it possible both to regulate the number of doctors and other working health professionals. It also allows assessing the training quality and ensuring respect for the technical and ethical aspects of their job [3].

Similarly, standards and procedures for recruiting staff in health structures exist and are generally applied by the regulation bodies: either the Ministry of the Public Service, the Ministry of Planning or the Ministry of Health itself [6].

### Human resource development in the DRC health system

In the Democratic Republic of Congo, human resource development is one of the six axes of the strategy for strengthening the health system adopted since 2006 and revised in 2010, and a document on staff standards in health districts has been drawn up [7–9]. This strategy is operationalized by a health development plan. The sector diagnosis of the 1st and 2nd edition plans identified the main priority problems of human resources for health, in particular the imbalance in the production and inequitable distribution of Human Resources for Health, the low motivation and loyalty of health personnel, the insufficient quality of education for health professionals and the poor development of the skills of health personnel [10, 11]. A national plan for the development of human resources for health has been drawn up in response to the problems identified. This plan aimed at “providing the health sector multidisciplinary, competent, high-performance health teams at all levels of the health pyramid, sufficient quantity and equitably distributed, contributing to the improvement of the state of health of the Congolese population through the provision of quality health care services”. One of the proposed solutions is the establishment of a health information system on HRH and the national observatory of HRH. The latter already exists, but is not documented [12].

Human resources are an essential pillar of the health system especially for countries in crisis such as the DRC. They allow the health system to function at its best despite the crisis context. Several authors have shown how a staff could help reduce the adverse effects of the crisis on the health system [2, 13–15].

As in most of African countries, the organization of the health system in the DRC is of the pyramidal type and includes three levels: the central level (National Ministry of Health), the intermediate level (Provincial Health Department) and the operational or peripheral level (the Health District) [7–11].

### Context of instability and its impact on the health system

Two billion people now live in situations of fragility and conflict [16]. The share of people living in extreme poverty in conflict situations is expected to rise from 17% of the global total to nearly 60% by 2030. More than a third of maternal deaths occur in fragile states, and half of all children who die before the age of five live in situations of fragility and conflict [17].

The WHO estimates that poor quality of care accounts for 15% of all deaths in low- and middle-income countries; and most likely even more in fragile, conflict-affected areas. The same source estimates that 60% of preventable maternal deaths, 53% of under-five deaths and 45% of neonatal deaths occur in fragile areas.

Many states classified as fragile are also in a post-conflict situation [18], which means that these countries have had to endure the destruction of their service infrastructure, which has worsened their service delivery situation.

Witter and Pavignani [19] have demonstrated the extent of conflict and state fragility and their negative impact on the functioning of the health system. They highlighted, among other things, the inability to provide health services to a large proportion of the population, the lack of political mechanisms to develop, establish and implement national health policies, inadequate capacity and management systems (such as budgeting, accounting and human resource management systems) to mobilize and control resources [19].

In the book on sustainability of health systems in crisis situations, Witter and Hunter argued that populations living in fragile, conflict-affected or vulnerable areas are at risk of worsening health conditions due to lack of access to even routine health care and the additional health risks associated with damaged infrastructure, physical and psychological trauma, and difficult living and economic conditions. Over 80% of major infectious disease outbreaks occur in these areas. Conflict areas, in particular, may face disruption of systems processes such as supply and health information, as well as the emigration or death of health workers.

There are also likely to be significant financial and resource constraints in these contexts that could impact on the quality of care in the health sector. The availability of an adequate workforce, both in terms of quantity and skills needed, is a common challenge [20].

Governments are therefore expected to do more with less, and it is the health workers who suffer the consequences of these pressures. These problems are compounded when some social groups perceive government as inefficient or not meeting expectations and thereby undermine its legitimacy, [20].

However, financing is only one of many important inputs in terms of health system sustainability. Yet human resources are also important and the targets for health systems to achieve Sustainable Development Goal 3 (SDG3) include one on recruitment, training and retention of staff [20].

The literature also indicates that some countries have taken advantage of the experience of the crisis to try to reorganize their health systems. The experience of the response to the Ebola virus disease (EVD) epidemic in Guinea provided an opportunity to reorganize the health system by investing in the workforce. A post-Ebola study provided strategic guidance to support the retention of health workers in rural areas [15].

### Context of instability and its impact on the health system in eastern DRC

The DR Congo has just completed three decades of crisis and instability. Eastern DRC was the first part to be affected by the crisis with the first Rwanda war in 1994, which led to the Rwandan genocide and dumped 1 million of the refugees in the two Kivu provinces [21]. Other crisis events have followed one another (the 1998 war, the Province crisis, the 2004 crisis in South Kivu, the Kasaï Oriental crisis and various movements of insecurity observed in various regions). Thus, Eastern DRC has been considered by some authors as the region at high risk of death, with the highest mortality rate since the Second World War. This crisis, whose number of deaths was initially estimated at 3 million in 2002 [22], woke up various specialists in armed conflict situations. A second study estimated the number of deaths linked to this crisis at 5 million [23]. The country is currently considered as an “unstable country” or “fragile state”, and some authors now speak of “mega crisis” [24].

Eastern DRC is still considered as a red zone and some foreign countries do not allow their citizens to visit the region despite the presence of MONUSCO for more than 15 years [25, 26]. The province of South Kivu is among the three most affected provinces, after North Kivu and Tanganyika. The displacement of populations due to the intensification of violent inter-community conflicts, combined with the looting of healthcare institutions, have contributed to creating a volatile situation that has led to the flight of qualified health workers from the concerned areas [20].

A major feature of the province of South Kivu in this decade of war is the looting of health infrastructure, particularly hospitals and health centers, by armed gangs. This situation has put hospitals and health centers in very difficult conditions for their operation [28].

This situation has plunged hospitals and health centers into very difficult conditions for their operation. Furthermore, the coordination structures (Provincial Health Inspectorate, Health District Offices and Central Health districts Offices) are only functioning at a minimum due to the lack of operating costs and logistical means to ensure their regulatory and supervisory role. The health personnel have become demotivated as a result of the non-payment of salaries and the lack of bonuses [28].

During these various crises, the health system was supported by both international partners and local organizations. Support from the health system was sometimes directed towards the rehabilitation of infrastructure, the supply of equipment and other inputs. This support was sometimes as subsidization of health care for the indigent and displaced populations, or direct remuneration of staff in the form of bonuses [10, 11, 23–25].

At the provincial level, the Provincial Health Department (PHD) of South Kivu has grouped health districts into three categories in 2010 and 2015, according to a number of criteria: developing health districts, health districts in transition and emergency health districts. This categorization included social, economic and political conditions; insecurity or armed conflict, geographical accessibility, etc. [28].

The health institutions involved in the provision of care are either public or private, or they depend on faith-based networks. Two of these faith-based networks are predominant, namely the network of the Catholic Church through the Diocesan Office of Medical Works and the network of the Protestant Church. Although they have a monopoly on the management of health resources who are registered to the National Public services and enjoy the same benefits as those in the public sector in accordance with the memorandum of understanding signed by the Ministry of Public Health [7, 11, 24]. With the new reform of the intermediate level, six working groups have been set up within the PHD, including the human resources working group, which normally has to analyze all the problems related to HRH and propose solutions [10, 28] but up to now this commission is not operational.

### Current evidences and debates on HRH in South Kivu

The issue of human resources could only arise when people have to be assigned or decommissioned by the political authorities.

Some of the current debates in Human Resources of Health (HRH) are dominated by the relationship between HRH and the state-building, particularly in fragile settings and after conflict [12, 18, 27].

Studies conducted on fragile settings and after conflicts zones such as DR Congo has globally highlighted the fact agents that work in urban and semi-urban benefit from more advantages than those in rural areas [38, 41]. None of them has deeply focused on fragile and post-conflicts Provinces such as South Kivu where armed conflicts have affected Health services settings.

Moreover, these studies have been conducted before the DR Congo to adopt the Human Resources Management as one of the strategic pillar of the Strengthening Strategy System for Health.

As long as debate on Human Resources for health and its role in state-building, many questions are possible. What is the case of rural zones commonly affected by conflicts after the DR Congo adopt that strategy? In a state-building point of view, did this strategy enhance the performance of Human Resources from recruitment to retirement especially in fragile and conflicts affected provinces such as South Kivu?

The context of fragility aggravates the underperformance of health systems as a whole, especially in low-income countries [19, 20].

The area of human resources management for health is among those most affected by the non-application of the usual policies and practices related to its management; thus, there is often an unavailability of qualified and motivated personnel, especially in rural areas, which leads to the dysfunction of health services [11, 38, 41].

According to WHO (2010), salaries are an important determinant of the quantity, distribution and performance of health workers. Low salaries for some categories of health workers can be a disincentive to enter the health professions or to accept a rural posting and do not motivate improved performance or quality.

Low salaries also affect several cross-cutting issues, including multiple job holding and migration to countries where salaries are much higher [41].

In the DRC, although Human Resource Management for Health has been adopted as one of the strategic axes of the SRSS by the Ministry of Health, this area remains a very weak link in the health sector, particularly in the health zones of South Kivu province (East of the country), which are in a fragile context.

It remains essential to guarantee the quality of health services to the population, especially in situations of permanent fragility such as in the east of the DRC. It therefore seemed very important to us to analyze the capacity of the Ministry of Health to continue to exercise its leadership in the management of HRH through the availability in crisis districts of personnel with an acceptable profile, meeting the standards and enjoying the regalian advantages for their motivation. It is impossible to make the health system effective without the availability of qualified and motivated HRH.

Our study aimed to answer the following questions:

- What is the profile of human resources for health in health districts in the context of conflict and ongoing fragility?
- What are the human resources for health factors associated with poor performance of health districts in conflict situations?

So, we aimed to: (1) analyze certain socio-demographic and economic characteristics such as age, gender, level of education and monthly income of the staff; (2) evaluate the recruitment and hiring process of health personnel as well as their distribution in the health zones targeted by the study; (3) evaluate the system of remuneration, payment of social benefits as well as the mechanization of the civil service of the agents in the health zones in crisis targeted by the study. The analysis of the level of performance of each health district associated with the profiles of the available HRH allowed us to answer the second question.

These analyses are an essential way of identifying the strategies and adaptations needed to maintain the proper functioning of the health system in a crisis context.

## Methods

### Study design and study population

A cross-sectional study was carried out among 1090 healthcare workers (medical, paramedical, administrative, and support staff) in 128 healthcare facilities in the four targeted health districts (Idjwi, Katana, Walungu and Mulungu). These facilities were categorized by the Provincial Health Department into stable, transitional and unstable health districts. A total of 1150 respondents were expected in the targeted districts and were distributed as follows: 280 for the Idjwi HD, 364 for Katana, 165 for Mulungu and 341 for the Walungu HD. Thus, the health personnel of 128 facilities, including 124 public, church and private integrated health facilities and 4 central offices of the health districts were considered in this study.

Of 1150 expected respondents, 1090 were approached, 1081 accepted to answer and only 9 refused to answer our questionnaire for personal reasons.

The Idjwi health district was considered as the most stable health district in South Kivu where no displaced persons and armed gangs are observed. This district is located in Idjwi territory and is accessible by lake.

The Mulungu Health district (HD) is the most unstable in the province. It is located in Shabunda territory where internally displaced persons and crisis events are at the upper level of the province. Accessibility in this HD is difficult by road and air [30].

Walungu and Katana HDs are intermediate districts. These districts have never experienced major crises or are post-conflict (in transition). Walungu is a post-conflict area and located in Walungu territory. Katana HD is an area that has never experienced major crisis and it is located in Kabare Territory with good accessibility by road.

### Data collection

An exhaustive paper survey was conducted by PHD investigators and trained data collectors in two stages: from May 15th to July 17th, 2017 for Katana and Walungu; from 1 April 1st to May 30th, 2019 for the Idjwi and Mulungu HDs.

This study included all health workers, doctors, nurses, laboratory technicians, nutritionists, pharmacists, administrative and support staff working in public, church and integrated private health facilities in the health districts of Idjwi, Katana, Mulungu and Walungu who agreed to participate in our study and are still actively working. All respondents gave an informed consent and agreed that they will be kept anonymous for confidentiality.

When the respondent was absent, the interviewer returned the next day.

Health care workers on layoff, on leave to absence, posted or under suspension were excluded from this study.

The number of agents is listed in Table 1 below. The health personnel surveyed all responded to our survey questionnaire. Before they respond to the questionnaire, they were told that their answers will be kept anonymous and that this study will be used for identifying the strategies and adaptations needed to maintain the proper functioning of the health system in a crisis context.

**Table 1:** Characteristics of rural health districts, eastern DR Congo, years 2017 to 2019

	Health districts				Standard
Features	Idjwi	Katana	Walungu	Mulungu	
Territory	Idjwi	Kabare	Walungu	Shabunda	
Population	294,209	236,986	285,669	170,439	≥ 100 000
Area (sqkm)	681	400	800	6.500	
Density (inhabitants/sqkm)	432	592	357	26	
Number of health areas	21	18	23	20	10–20
Number of hospitals	4	2	3	2	1
Number of other agents	280	364	165	341	
Number of health centers	21	18	23	20	
Health coverage (CS /10,000 hab) %	72%	75%	79%	117%	≥ 80%
Number of doctors	8	10	9	7	
Number of nurses	150	166	179	98	
Number of doctors /10,000 hab	0.27	0.42	0.31	0.41	1/10000
Number of nurses / 5000 hab	2.54	3.53	3.14	2.88	1/5000
Manager	BDOM^a^	BDOM	State	State	
Partner	BDOM, AAP, IRC^b^	BDOM, AAP, LC^c^	LC, AAP	MSF^d^, ICRC^e^	
Crisis situation	Stable	Post-crisis	Post-crisis	In crisis	
Performance of health zones (%)	75.3*	79.9*	58.6*	52.3*	≥ 75%
Average performance of the Provincial Health Division of South Kivu for the three years covered by the study				66.1	

*Average performance of health zones for the three years concerned by the study (2017–2018–2019). ^a^BDOM = Bureau Diocésain des Œuvres Médicales (The Diocesan Office of Medical Works), Catholic faith-based. ^b^IRC = The International Rescue Committee. ^c^LC = Louvain Cooperation. ^d^MSF = Médecins Sans Frontières (Doctors Without Borders). ^e^ICRC = International Committee of the Red Cross. AAP = Agence d’Achat des performances

To categorize the agents of different study areas, we used the classification of agents according to the public services of the Congolese State which distributes agents according to the following categories: Category A (senior officials): Secretary General, Director General, Director; Category B (senior managers): Head of Division and Head of Office; Category C (collaboration agents): 1st Class Administrative Attaché, 2nd Class Administrative Attaché and 1st Class Administrative Agent and Category D(executing agents): 2nd Class Administrative Agent, 1st Class Auxiliary Agent, 2nd Class Auxiliary Agent and the Bailiff.

The performance was defined in accordance with DRC national guidelines related to indicators of performance. For the HD performance, we used routine collected data where the Health District Performance is assessed by an assessment tool. This assessment tool includes six indicators and is used for routine data collection for Health District performance assessment.

### Data processing and analysis

We used the Excel program for data entry. The data were then exported to Epi info 7 and SPSS 25 for processing and analysis.

The quantitative variables were described by their median [median (interquartile range)] following their asymmetric distributions and the qualitative variables in absolute and relative frequencies. The Chi-square test was used for the comparison of proportions and the Kruskal–Wallis test for medians.

To describe the specific characteristics of the different health districts, we made different comparisons between the zones according to the context of the crisis. As a measure of association, we used unadjusted odds ratios (UORs) with their 95% confidence intervals. We then built multivariate logistic regression models to determine the factors associated with the poor performance of health zones. Adjusted odds ratios were generated along with their 95% confidence intervals. The α-error cut-off was set at 5%.

## Results

### Profile of health care workers

It emerges from this table that health coverage is beyond standards (≥ 80%) than in the unstable health districts of Mulungu. It is lower in the Idjwi stable district. This area is still the one with a high number of doctors per 10,000 inhabitants compared to the others despite no area approaching the standards (i.e., at least one doctor per 10,000 inhabitants).

For the number of nurses, all these areas have an average of 3 nurses per 5000 inhabitants, which is in line with the standards which foresee at least one nurse for 5000 inhabitants.

Two health districts performed very well, namely the Idjwi zone and the Katana district. The areas with good performance all have in common the Performance Purchasing Agency as Partner (AAP) and are all under the management of the Catholic Church. The Idjwi area is stable and Katana in post-crisis. The areas with poor performance are areas under state management; these are Mulungu and Walungu. Walungu is a post-crisis area and Mulungu is in crisis. The province’s average performance over the study period is poor.

Table 2 shows the socio-demographic characteristics of the population. In all health districts, subjects over 34 years old were the most represented. The median age was 46 years, the oldest health worker was 84 years old and he was from Mulungu HD. The youngest was 20 years old. Women were the less represented in all health districts especially in Mulungu (23%). 44% of the agents had a high level of education (university or high institute). 68% the Idjwi health district had a high level of education, followed by Mulungu health district (60%). In all health districts, the nursing/medical staff (47%) was less represented than the administrative staff (53%).

**Table 2:** Socio-demographic characteristics of the respondents according to rural health districts, eastern DR, Congo, years 2017–2019

Variables	ALL *n* (%)	Mulungu *n* (%)	Idjwi *n* (%)	Katana *n* (%)	Walungu *n* (%)
Age (years)	46 (20–84)*	43 (20–84)*	40 (22–75)*	50 (20–81)*	49 (20–81)*
≤ 34	277 (26)	32 (21)	97 (37)	72 (22%)	76 (24)
> 34	783 (74)	124 (79)	162 (63)	260 (78%)	237 (76)
Gender					
Male	737 (68)	120 (77)	192 (69)	211 (64)	214 (68)
Female	341 (32)	35 (23)	86 (31)	121 (36)	99 (32)
Level of education					
< Superior	601 (56)	66 (40)	88 (32)	215 (65)	232 (74)
Superior	480 (44)	98 (60)	184 (68)	117 (35)	81 (26)
Facilities					
HDCO	52 (5)	7 (5)	10 (4)	16 (5)	19 (6)
Hospital (RH + HC)	372 (35)	43 (28)	103 (37)	117 (35)	109 (35)
Health center	649 (60)	102 (67)	163 (59)	199 (60)	185 (59)
Status of the facilities					
Public	684 (65)	142 (99)	144 (55)	192 (64)	206 (60)
Other	364 (35)	1 (1)	116 (45)	107 (36)	140 (40)
Marital status					
Living alone	189 (17)	28 (18)	51 (17)	56 (17)	54 (17)
As a couple	919 (83)	129 (82)	255 (83)	276 (83)	259 (83)
Seniority (years)	10 (0–59)*	7 (0–19)*	8 (0–35)*	13 (2–59)*	13 (2–59)*
≤ 4	289 (28)	61 (44)	90 (34)	73 (22)	65 (21)
> 4	758 (72)	79 (56)	172 (66)	259 (78)	248 (79)
Family residence					
In the working area	953 (88)	137 (86)	238 (87)	301 (91)	277 (88)
Out of working area	126 (12)	22 (14)	37 (13)	31 (9)	36 (12)
Position in the facilities					
Administrative officer	571 (53)	84 (53)	130 (47)	168 (51)	189 (60)
Medical doctor and nurse	509 (47)	76 (48)	145 (53)	164 (49)	124 (40)

*Median (min–max), primary and secondary level, RH = referral hospital HC = hospital center, HDCO = Health District Central office, *n* (%) = number (percentage)

### Recruitment, hiring and promotion process

Table 3 shows that 63% of agents do not have State registration numbers.

**Table 3:** Administrative status, history of recruitment and promotion in four rural health districts in eastern DR Congo, 2017–2019

Variables	All *n* (%)	Mulungu *n* (%)	Idjwi *n* (%)	Katana *n* (%)	Walungu *n* (%)
Rank*					
Category c	417 (40)	59 (37)	95 (35)	121 (39)	142 (47)
Category d	628 (60)	101 (63)	175 (65)	190 (61)	162 (53)
Status in public service					
Registered state agent	391 (37)	79 (49)	119 (46)	117 (35)	76 (24)
Non-registered state agent	675 (63)	82 (51)	141 (54)	215 (65)	237 (76)
Recruitment authority					
Central and intermediate level	629 (59)	120 (73)	85 (32)	225 (68)	199 (64)
Other	442 (41)	44 (27)	177 (68)	107 (32)	114 (36)
Recruitment process					
Job application	491 (46)	18 (11)	158 (59)	173 (52)	142 (45)
Other	588 (54)	146 (89)	112 (41)	159 (48)	171 (55)
Continuing education					
Have continuing education	205 (49)	55 (36)	150 (56)	129 (41)	119 (36)
Do not have continuing education	216 (51)	99 (64)	117 (44)	184 (59)	213 (64)
Promotion since enlistment					
Yes	121 (28)	47 (29)	74 (27)	88 (28)	113 (34)
No	313 (72)	117 (71)	196 (73)	225 (72)	219 (66)

*According to the categorization of agents in the public service of the DRC (explanation in the methodology)

### Remuneration and payment of staff benefits

Table 4 shows that 95% of health personnel in all health districts do not receive state salary and 66% do not receive the Government bonus. However, 88% of health care workers receive the local bonus and 63% of agents do not have State registration numbers.

**Table 4:** State salary, local salary and risk allowance of the respondents according to the rural health districts, eastern DR Congo, years 2017–2019

Variables	ALL *n* (%)	Mulungu *n* (%)	Idjwi *n* (%)	Katana *n* (%)	Walungu *n* (%)
Receive state salary					
Yes	50 (5)	5 (3)	11 (4)	18 (6)	16 (5)
No	1022 (95)	157 (97)	254 (96)	295 (94)	316 (95)
Receive local bonus					
Yes	955 (88)	83 (52)	273 (99)	304 (97)	295 (89)
No	126 (12)	76 (48)	4 (1)	9 (3)	37 (11)
Receive the government bonus					
Receive	356 (34)	64 (39)	94 (35)	18 (5)	197 (64)
Do not receive	706 (66)	99 (61)	177 (65)	318 (95)	112 (36)

*According to the categorization of agents in the public service of the DRC (explanation in the methodology), *n* (%) = number (percentage)

The characteristics of agents working in areas with poor performance are [for any characteristic we give the Unadjusted odds ratio (UOR) of the category in relation to the poor performance, its confidence interval (IC à 95%) and the degree of significance (*P*)], the age of the agents {greater than 34 years [UOR (IC à 95%) = 1.3 (1.01–1.77), *P* = 0.040)]}, their level of education {the low level [UOR (IC à 95%) = 1.6 (1.3–2.1), *P* < 0.001]}, the status of the facilities {Public [UOR(IC à 95%) = 1.6 (1.3–2.1), *P* < 0.001]}, the position in the facilities {administrative officer [UOR (IC à 95%) = 1.4 (1.1–1.8), *P* = 0.005]}, the category according to the public services of the Congolese {Category c [UOR(IC à 95%) = 1.3 (1.103–1.7), *P* = 0.044]}, the status in public service {non-registered state agent [UOR (IC à 95%) = 1.4 (1.1–1.8), *P* = 0.015]}, the recruitment process {recruitment processes other than job application [UOR (IC à 95%) = 2.4 (1.9–3.1), *P* < 0.001]}, the continuing education {the agents who do not have continuing education [UOR (IC à 95%) = 1.7 (1.3–2.1), *P* < 0.001]}, receive local bonus { no receive local bonus [UOR (IC à 95%) = 13.3 (7.4–23.9), *P* < 0.001]} (Table 5).

**Table 5:** Relationship between staff profile and the performance of health districts, eastern DR Congo (univariate and multivariate analysis), years 2017–2019

Variables	HD with poor performance	HD with Good performance	Unadjusted OR (IC à 95%)	*P*	Adjusted OR (IC à 95%)	*P*
Age (years)						
≤ 34	108	169	1		1	
> 34	361	422	1.3 (1.01–1.77)	0.040	1.6 (0.5–1.9)	0.066
Gender						
Male	334	403	1.3 (0.9–1.7)	0.063	1.1 (0.7–1.6)	0.120
Female	134	207	1		1	
Level of education						
< Superior	298	303	1.6 (1.3–2.1)	< 0.001	1.8 (1.3–2.4)	< 0.001
Superior	179	301	1		1	
Facilities						
HDCO	26	26	1.4 (0.8–2.6)	0.211	1.2 (0.1–4.1)	0.099
Hospital (RH + HC)	152	220	1		1	
Health center	287	362	1.1 (0.9–1.5)	0.296	1.2 (0.4–1.9)	0.390
Status of the facilities						
Public	348	336	1.6 (1.3–2.1)	< 0.001	2.1 (1.1–3.2)	0.021
Private	141	223	1		1	
Marital status						
Living alone	82	107	1.0 (0.8–1.4)	0.768		
As a couple	388	531	1			
Seniority (years)						
≤ 4	126	163	1		1	
> 4	327	431	0.9 (0.7–1.3)	0.893	0.6 (0.4–1.9)	0.690
Family residence						
In the working area	414	539	1			
Out of working area	58	68	1.1 (0.8–1.6)	0.582		
Position in the facilities						
Administrative officer	273	298	1.4 (1.1–1.8)	0.005	1.7 (0.8–2.1)	0.063
Medical doctor and nurse	200	309	1		1	
Rank*						
Category c	201	216	1.3 (1.03–1.7)	0.044	1.5 (0.9–2.1)	0.055
Category d	263	365	1		1	
Status in public service						
Registered state agent	155	236	1		1	
No-registered state agent	319	356	1.4 (1.1–1.8)	0.015	1.1 (0.8–2.2)	0.079
Recruitment authority						
Central and intermediate level	319	310	1		1	
Other	158	284	0.5 (0.4–0.7)	< 0.001	0.8 (0.6–1.1)	0.059
Recruitment process						
Job application	160	331	1			
Other	317	271	2.4 (1.9–3.1)	< 0.001	2.2 (1.8–4.1)	< 0.001
Continuing education						
Have continuing education	174	279	1			
Do not have continuing education	312	301	1.7 (1.3–2.1)	< 0.001	1.9 (1.2–2.6)	< 0.001
Promotion since enlistment						
Yes	160	162	1		1	
No	336	421	0.8 (0.6–1.0)	0.110	1.1 (0.9–1.5)	0.212
Receive state salary						
Yes	21	29	1			
No	473	549	1.2 (0.7–2.1)	0.553		
Receive local bonus						
Yes	378	577	1			
No	113	13	13.3 (7.4–23.9)	< 0.001	6.1 (4.2–20.1)	< 0.001
Receive the government bonus						
Receive	261	112	1			
Do not receive	211	495	0.8 (0.6–1.0)	0.058		

*RH* referral hospital, *HC* hospital center, *HDCO* Health District Central Office, *n* (%) number (percentage), *HD* health zone, *UOR* unadjusted odds ratio**,** *AOR* adjusted odds ratio

After adjustment, the characteristics of agents working in underperforming areas remain [for any characteristic we give the adjusted odds ratio (AOR) of the category in relation to the poor performance, its confidence interval (IC à 95%) and the degree of significance (*P*)], the continuing education {the agents who do not have continuing education [AOR(IC à 95%) = 1.9 (1.2–2.6), *P* < 0.001)]}, their level of education {the low level [AOR(IC à 95%) = 1.8 (1.3–2.4), *P* < 0.001])}, the Status of the facilities {Public [AOR(IC à 95%) = 2.1 (1.1–3.2), *P* = 0.021]}, the recruitment process {recruitment processes other than job application [UOR(IC à 95%) = 2.2 (1.8–4,1), *P* < 0.001]}, receive local bonus { no receive local bonus [UOR(IC à 95%) = 6.1 (4.2–20.1), *P* < 0.001]} (Table 6).

**Table 6:** Monthly income by occupation and level of education of respondents of the health districts in the context of instability, eastern DR Congo, years 2017 to 2019

Monthly income by function held in the structure ($)					
	Median (Min–Max)				
	Mulungu	Katana	Walungu	Idjwi	*P*-value*
Medical doctor	167 (55–355)	217 (60–779)	387 (83–805)	273 (167–1033)	< 0.001
Administrative	51 (5–90)	30 (7–211)	40 (7–250)	40 (4–402)	0.050
Nurses	52 (6–115)	50 (7–1000)	63 (10–250)	70 (7–183)	< 0.001

*Kruskal–Wallis

Table 7 compares the median monthly salaries of health zone personnel in the different zones studied according to the type of agents. It revealed that overall; the median of agents’ salaries is statistically different according to the type of agents (< 0.001). Doctors and nurses have different median wages (< 0.001) in the different areas studied. Doctors in Mulungu HD have the lowest median salary of all areas whereas the highest doctor’s salary is found in Walungu Health District.

**Table 7:** The factors associated with the poor the performance of health districts in the context of instability, eastern DR Congo (multivariate analysis), years 2017 to 2019

Variables	Adjusted OR (IC à 95%)	*P*
Do not have continuing education	1.9 (1.2–2.6)	< 0.001
Low level of education	1.8 (1.3–2.4)	< 0.001
Recruitment process other than job application	2.2 (1.8–4.1)	< 0.001
No receive local bonus	6.1 (4.2–20.1)	< 0.001
The public status of the facilities	2.1 (1.1–3.2)	0.021

*AOR* unadjusted odds ratio

**Fig. 1:**
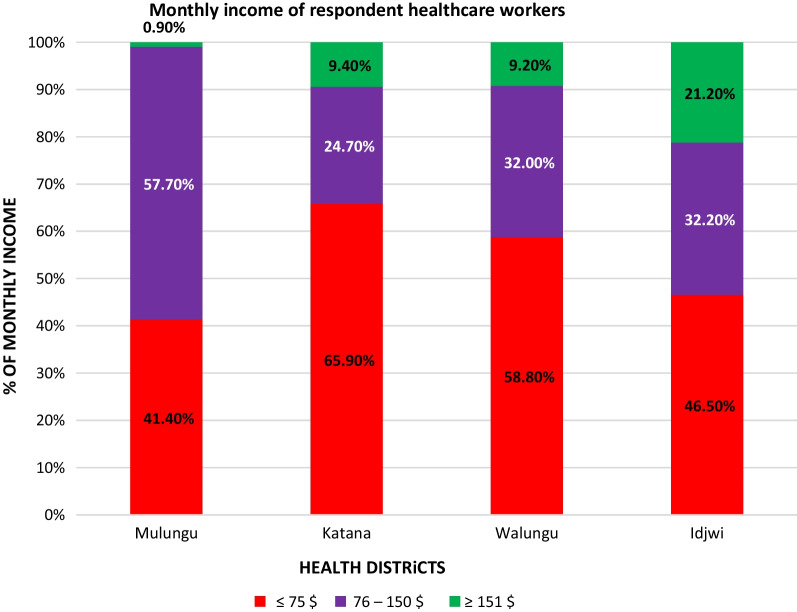
Monthly income of respondents by health districts, eastern of DR Congo, years 2017 to 2019 (Chi-square test, *P* < 0.001)

These results in Fig. 1 indicated that it was in the Idjwi Health District (stable) where we found a high proportion (21.20%) of health workers with a monthly income greater than or equal to $151 compared to other health districts (*P* < 0.001). In the Mulungu Health District (unstable), their monthly income was very low (0.90% of < $151) versus 9.4% in the Katana Health District and 9.2% in the Walungu Health District (in transition).

## Discussion

This study describes the profile of the staff in the rural health districts of South Kivu, in the east of the Democratic Republic of Congo. More specifically, the objectives were to assess (1) the profile of the staff, (2) the recruitment and hiring system, (3) the remuneration system and payment of allowances.

### Socio-demographic and economic characteristics of the respondents according to the health districts studied

This study showed a statistically significant difference for age of the staff in the studied health districts. The Idjwi HD (stable district) has a lower median age [40 (22–75) years] compared to other HDs. We also noted that the Katana HD has a large proportion of staff who are already over the retirement age of 65 years (23.8%), followed by the Walungu zone (19.2%) compared to the Idjwi HD (4.2%) and the Mulungu HD (3.2%).

These results are not consistent of those found by Patrick Ilboudo et al. [35], in the 2014 Burkina Faso Health Personnel Survey, regarding the age of health personnel that ranged between 27 and 57 years old. Gautier and Wane in Chad where the majority of agents were between 31 and 45 years old [36]. This can be explained by the fact that the retirement system has not been functional in the DRC for several years.

Concerning the seniority of health staff in the study areas, it was found that staff in Mulungu had a younger seniority [7 (0–19) years] than in Idjwi [8 (0–35) years], Katana and Walungu [13 (2–59) years] and difference was statistically significant (*P* < 0.001). The results also show that Walungu HD had a high proportion (8.9%) of staff above the retirement age ≥ 65 years for seniority, followed by Katana (7.2%) compared to Idjwi (0.3%) and Mulungu (0.0%).

These findings are consistent with a study conducted in Papua New Guinea on rural health workers and their work environment [37], in which most respondents had at least six years of nursing experience (44%), and the number of years in the profession was significantly associated with job satisfaction (*P* < 0.05). Patrick Ilboudo et al. [35], also show in their study that the health personnel concerned had a public work experience of 115 (10–412) months, corresponding to about 10 years, and a job experience that varied between 16 and 116 months. Rishma et al. [38], show that the majority of health workers in the DRC had a median seniority of 6 years.

From these results, we find that in Mulungu, staff appear older than in Idjwi and other areas, but for seniority, it is in Mulungu where the observed age is younger than elsewhere. This implies that Mulungu recruits more elderly people than Idjwi and the zones. This would be explained by the fact that Mulungu is a very unstable area due to armed conflicts, and that as young people are generally more likely to leave the area in search of places with better security conditions, older people find themselves competitive in this area.

Our study showed that in the Idjwi HD (stable district) a high proportion of staff had a higher level of education than in the transitional/conflict HDs (67.6% in Idjwi compared to 59.8% in Mulungu, 35.2% in Katana and 25.9% in Walungu) with a statistically significant difference in education level (*P* < 0.001).

A cross-sectional study of the sources of income of frontline health workers in the Democratic Republic of Congo [41], shows that the majority of health workers are at secondary level, only 30% had a higher education level and university. The age ranged from 30 to 40 years, 90% of the staff were nurses and only 4% were doctors.

Regarding the status of the structures in the study districts, comparing the Idjwi Health District (stable district) with other districts, we find that there is a statistically significant difference between the Idjwi Health District and the Mulungu and Walungu Health districts (*P* < 0.05). Here, 100% of the structures in the Mulungu HD (unstable/conflicted) are state-owned. This could be explained by the fact that private and religious organizations always choose to invest in stable, secure environments where the population has an economic level capable of meeting the cost of care.

Joyasuriya et al. [37] in their study show that 50% of the respondents worked in government facilities and 40% in church-owned facilities, compared to only about 3% in the private sector.

### Assessment of the recruitment and hiring process of health personnel and their distribution in the targeted health districts

Our study shows an insufficient number of medical doctors compared to the Ministry of Health’s standards. In the DRC there should be at least 1 medical doctor for every 10,000 inhabitants, however in the health zones of the study we observed an average of 0.35 doctors for the same population [9]. The average of this ratio in South Kivu province is 0.7 and that of the DRC country is 0.9 [30]. On the other hand, an average of 3 nurses per 5,000 inhabitants is observed, which is within the norms that require at least one nurse for the same number of inhabitants.

Indeed, more than half of the doctors are concentrated in urban health districts where living conditions and security are acceptable with the possibility of offering paid services in private clinics and thus having an increase in their income. The WHO in its World Health Report 2006 reveals similar geographical disparities in other parts of the world [1]. In the case of the province studied, factors related to permanent states of insecurity do not encourage doctors to go to very remote areas, not only because of the precarious living conditions but also because they are often targeted by armed gangs [28, 32].

On the other hand, the sufficient number of nurses in the DRC and particularly in the health districts studied could be explained by the multiplication of nurse training schools in rural areas. Indeed, for electoral reasons, most politicians have negotiated and obtained the opening of nursing schools and training institutes in their localities, but the quality of training remains low. Indeed, according to the study by Wabasa et al. (2017) several problems however can be associated with poor governance in the higher education sector in DR Congo. The most critical ones can be summarized as follows: (1) the prevalence of informal management, (2) the grip of politics on the higher education sector (its politicization).

This study shows a disproportion between the number of nurses and administrative staff of 47% and 53%, respectively, which is outside the norms of the DRC Ministry of Health, which recommends 70% nurses against 30% administrative staff [9]. The WHO World Health Report makes the same observation for the majority of low-income countries [2]. In the case of the DRC, this is because the government does not retire workers, as ageing workers can no longer work, and others are recruited in their place, but the former are still on the list and continue to report to the service in order to retain their benefits, still hoping to retire.

The results of the study show that 47% of the staff are administrative personnel. Doctors and nurses are more present in the stable district than in the intermediate district. The results of our study differ slightly from the results of the 2015 country profile study on human resources for health in the Democratic Republic of Congo [42], which found that at the national level, administrative staff represent 38.4%. Indeed, with the multi-faceted crisis in the east of the country, leaders are forcing the hiring of staff even when they are not needed; this is easier for administrative staff whose technical skills are often confused (jack of all trades).

### Evaluation of the remuneration system, payment of allowances and mechanization in the civil service

Our results show that medical doctors are the highest paid staff compared to other staff. They then show that the incomes of doctors and nurses are distributed differently in the study areas (*P* < 0.001). Our data are consistent with those of Patrick Ilboudo et al. [35] which also shows a difference in median income between staff according to the position held (*P* = 0.0321). Their study shows that the median income for all health staff is $295.2 ($59.4–797.4) whereas for doctors it is $369 ($59.4–646.2). Maria Paola et al. [41], also show in their study that in the DRC, the median monthly income of medical doctors is $785 with a maximum of $4815; administrative staff have $166 and a maximum of $1396 while nurses have a median income of $101 and a maximum of $2908. Rishma et al. [33], in the cross-sectional study of income sources of frontline health workers in the Democratic Republic of Congo, also shows that in the DRC, the monthly income from all sources was $85, but the average was almost double that ($165). The highest median monthly income was for non-clinical work outside the facility ($119), followed by government wages ($58). The lowest median monthly income was from per diems ($9) and informal payments ($9).

Our data show that there are more agents with more than $151 (21%) in the stable district, while in the intermediate district this proportion is less than 10% and less than 1% in the unstable district. This difference can be explained by the low use of health services by the population due to the rural exodus and wars. This would explain the maximization of low income in unstable or transitional districts [36].

In the case of this study, this difference is explained by a system of distributing staff bonuses according to the indexes given to each agent according to their level of education and professional category; as there are fewer doctors with a higher level of education, they also earn more money. Another element is that some NGOs that support these crisis health areas already set fixed amounts for the payment of staff and certain categories such as nurses are more favored to keep them in place. Given that state regulation is weak in these provinces and ‘in the name of peace’ given the still fragile context, this is allowed to pass.

As for other benefits, such as the granting of a risk premium, this was decided and distributed at national level, giving more to doctors (minimum of $500) and a little less to nurses (maximum of $120) and even less ($50) to administrative staff. The question of benefits is therefore not the prerogative of the operational level but rather of the national level, and the difference between the crisis zone and the post-crisis zone is not perceptible; it is the pressure of the unions of each corporation that is at stake to wrest these benefits from their union members. The same applies to the allocation of civil service numbers. It should be noted, however, that some local political decision-makers also use their powers to facilitate obtaining these benefits for their beneficiaries. For example, some officers are left without hazard pay or government salary because they have not been registered into the civil service for several years.

The results of the study show that compared to the status of civil servants (registered agent, New Unit, No status), registered agents are twice as likely to be in the stable district. Indeed, administrative procedures seem to be more respected. This is probably due to the context of insecurity, which leads to both the flight of agents and the use of available agents who have not yet completed the administrative formalities. A qualitative study of these agents and their employers would be necessary to understand why some workers in the transitional districts do not have a registration number.

The application of regulations relating to the recruitment process of health personnel, their distribution in health districts and the provision of services in unstable health districts remains possible but requires certain adaptations, notably an improvement in the level of governance.

### Human resource factors associated with the performance of health districts

The results of the study show that the human resources for health factors associated with the poor performance of the health districts are successively the lack of continuous training of staff, the low level of training of agents, belonging to a health district under state management, the non-respect of the recruitment process and the failure of agents to receive local bonuses. Studies conducted elsewhere [1, 4, 15, 26, 39] have also shown that very careful human resource management was associated with better performance. In implementing these interventions that would enable the achievement of the Millennium Development Goals, they emphasized the multiple problems faced by healthcare workers the main ones being Inadequate or insufficient training, with curricula that are not adapted to needs; lack of management attention; poor human resource development policies and practices (poor career prospects, working conditions and remuneration).Three categories of strategies can be put in place to address these problems [1]: (1) strategies that improve recruitment, (2) strategies that help existing staff perform better and (3) those that slow down attrition. These authors say that it is therefore the responsibility of countries and leaders at all levels to put good policies in place [1, 4, 15, 26]. In the case of our study, we unfortunately found that the fact that a health district is under direct state management is a factor associated with poor performance. According to our observations, this could be explained by the context of fragility and conflict in the region, making the role of regulation very difficult and complex; In the field, it was observed that in purely state health zones, the recruitment of personnel and their management in general are more tainted by subjectivity for the purposes of political arrangements (agreements for peace, geopolitical balance, imposition of a leader, etc.) and do not respect the process of regulation) and did not respect the process. In the health districts where management has been handed over to private religious organizations, these practices are less observed, given the reputation of the religious leaders who try to ensure that the procedures for managing personnel are respected.

### Limitations

Despite its proven merit, this study has some limitations. Firstly, a significant number of non-responses were observed during the interviews. Fortunately, this did not significantly impact the overall analyses. Secondly, the analysis on human resources was limited only to their use, which normally required studying production, use, and retirement as well. Thirdly, other aspects that seem to be important for human resources but not taken into account in this study are the level of satisfaction of health workers, retention, working conditions including benefits, housing, allowances, etc. Moreover, this study did not assess the consideration of other sources of income besides state salary, state and local bonuses, and finally the workers’ assessments of security crises and their impact on the performance of their tasks. Future studies that address this matter are needed.

## Conclusion

There is a shortage of medical staff and a high proportion of administrative staff compared to caregivers in all of the four rural health zones under study.

Specifically unstable (emergency) health districts use older staff with less seniority than other zones; there are also more men, staff with lower levels of education and most of them do not have a State registration number. Staff in stable health districts benefits more from continuing education (capacity building) than those in unstable (crisis) health districts, and in terms of salary, they earn more, with a high monthly income. Informal recruitment is associated to poor performance of the health districts.

Other studies would be more important to deepen the analysis on human resources by considering parameters not studied in this work, such as the production of human resources, the agents’ level of satisfaction and their attitude towards the security crisis.

The context of instability compromises the performance of the health system by depriving it of competent personnel, so the management of health care workers requires some adaptations.

The State should develop incentive mechanisms to motivate young and trained personnel to work in unstable health areas, including improving their living conditions and working conditions.

## Data Availability

The datasets used and/or analyzed during the current study are available from the corresponding author on reasonable request.

## Abbreviations

DR: Democratic Republic of
DRC: Democratic Republic of Congo
BDOM: Bureau Diocésain des Œuvres Médicales
EVD: Ebola virus disease
HD: Health district
HRH: Human resources for health
HDCO: Health District Central office
PHD: Provincial Health Department
IRC: International Rescue Committee
LC: Local committee
MSF: Médecins Sans Frontières
NGO: Non-government organization
RH: Referral hospital
HC: Health center
WHO: World Health Organization
